# Peptide Lv and Angiogenesis: A Newly Discovered Angiogenic Peptide

**DOI:** 10.3390/biomedicines12122851

**Published:** 2024-12-15

**Authors:** Dylan L. Pham, Kelsey Cox, Michael L. Ko, Gladys Y.-P. Ko

**Affiliations:** 1 Department of Veterinary Integrative Biosciences, College of Veterinary Medicine and Biomedical Sciences, Texas A&M University, College Station, TX 77843, USA; 2 Department of Medical Physiology, School of Medicine, Texas A&M University, Bryan, TX 77807, USA; 3 Department of Biology, Division of Natural and Physical Sciences, Blinn College, Bryan, TX 77802, USA; 4 Texas A&M Institute for Neuroscience, Texas A&M University, College Station, TX 77843, USA

**Keywords:** angiogenesis, neovascularization, peptide Lv, ion channels, vascular endothelial growth factor, VEGF

## Abstract

Peptide Lv is a small endogenous secretory peptide with ~40 amino acids and is highly conserved among certain several species. While it was first discovered that it augments L-type voltage-gated calcium channels (LTCCs) in neurons, thus it was named peptide “Lv”, it can bind to vascular endothelial growth factor receptor 2 (VEGFR2) and has VEGF-like activities, including eliciting vasodilation and promoting angiogenesis. Not only does peptide Lv augment LTCCs in neurons and cardiomyocytes, but it also promotes the expression of intermediate-conductance K_Ca_ channels (K_Ca_3.1) in vascular endothelial cells. Peptide Lv is upregulated in the retinas of patients with early proliferative diabetic retinopathy, a disease involving pathological angiogenesis. This review will provide an overview of peptide Lv, its known bioactivities in vitro and in vivo, and its clinical relevance, with a focus on its role in angiogenesis. As there is more about peptide Lv to be explored, this article serves as a foundation for possible future developments of peptide Lv-related therapeutics to treat or prevent diseases.

## 1. Introduction

Angiogenesis is the growth of blood vessels from pre-existing vasculature. This process is essential during development and wound healing [1,2], but it also contributes to the pathology of various diseases, including metastatic cancers [3,4], atherosclerosis [5], arthritis [6], and ocular diseases, such as diabetic retinopathy and age-related macular degeneration [7,8]. Therapies targeting angiogenic factors, such as the vascular endothelial growth factor (VEGF), are widely used to combat these diseases [9,10,11]. However, nearly 30% of patients do not respond or become resistant to anti-VEGF therapies for reasons that are still unclear [11,12,13]. Repeated anti-VEGF injections are needed to block recurring neovascularization, which cause further unwanted side effects [11,12,13]. A possible explanation for the resistance to anti-VEGF therapies and recurring neovascularization is the involvement of other angiogenic factors that are insensitive to anti-VEGF agents [11,12,13,14]. Thus, identifying these angiogenic factors and delineating their molecular mechanisms is clinically imperative for the development of new therapies against diseases involving pathological neovascularization.

## 2. Discovery of Peptide Lv and Its Bioactivities

The Ko laboratory discovered a small endogenous secretory peptide (~40 amino acids), named “peptide Lv” (human gene ID: 196740, a.a. 55–94; mouse gene ID: 320736, a.a. 55–103) [15]. In silico screening with human and mouse cDNAs was employed to identify novel secretory peptides that had not been characterized before [15].

After a list of candidates was chosen, they were tested for their ability to augment L-type voltage-gated calcium (Ca^+2^) channels (LTCCs) in photoreceptors, using patch-clamp recordings [15]. LTCCs are responsible for neurotransmitter release in retinal photoreceptors and synaptic plasticity in neurons [16,17,18]. Peptide Lv, indeed, augments LTCCs in cultured photoreceptors, hence its name “peptide Lv” [15].

**Figure 1:**
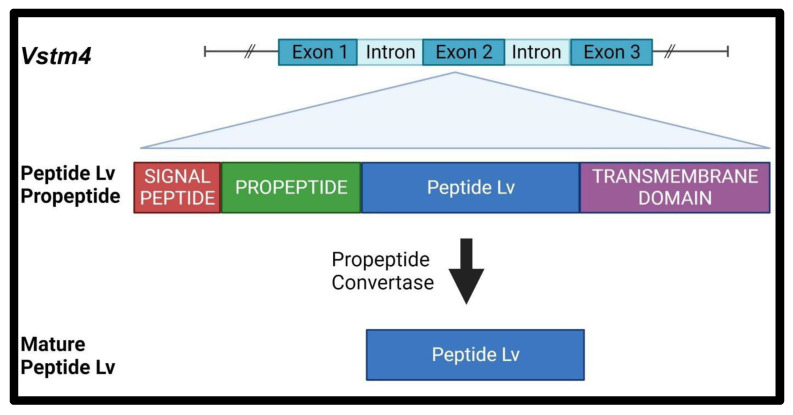
Peptide Lv is encoded as a propeptide in the *Vstm4* gene. Created with Biorender.com.

**Figure 2:**
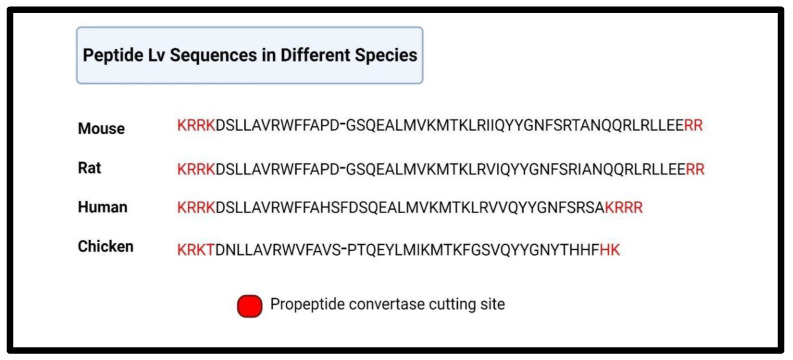
Sequence homology of peptide Lv among four different species: mouse, rat, human, and chicken. Created with Biorender.com.

Peptide Lv is encoded in exon 2 of the *Vstm4* (V-set transmembrane domain-containing 4) gene as a propeptide and contains a propeptide convertase cutting site and a secretory signal peptide domain (Figure 1). It is highly conserved among several species, including human, rat, mouse, and chicken (Figure 2) [15], and is widely expressed in various organs, including the brain, eye, heart, liver, lung, spleen, and intestines, and in vascular endothelial cells [15,19]. The mRNA of peptide Lv is present in specific brain areas, including the hippocampus, olfactory bulb, cerebellum, and cerebral cortex, and is expressed the most in the outer nuclear layer (photoreceptor layer) of the retina [15].

Cultured photoreceptors treated with peptide Lv have increased mRNA and protein expression of LTCCs (Cav1.2 and Cav1.3) and increased current densities [15]. In addition, photoreceptors treated with peptide Lv have increased levels of cyclic adenosine monophosphate (cAMP) and increased phosphorylation of extracellular signal-regulated kinase (ERK), two molecules involved in augmenting LTCC activities [20,21]. Interestingly, treatment with the pertussis toxin, a Gα-protein inhibitor, dampens the increase in cAMP levels and phosphorylation of ERK, but does not affect the peptide Lv-elicited increase in the LTCC current densities [15]. This result suggests that peptide Lv has a Gα-protein-independent pathway to augment LTCCs.

To determine the potential binding partners of peptide Lv, co-immunoprecipitation with a specific peptide Lv antibody, anti-Lv, was performed using whole mouse brain lysates and the protein bands with molecular weights between 40 and 250 kD were isolated for further identification [19]. Several binding partners were identified using mass spectrometry, including the kinase insert domain receptor family (KDR) that comprises the VEGF receptor 2 (VEGFR2), Fc receptor-like B (FCLRB), and vomeronasal type-1 receptor 1 [19]. VEGF binding to VEGFR2 promotes angiogenesis during development and wound healing and plays an important role in regulating the cardiovascular system [22]. FCRLB is a member of the Fc receptor family, which is involved in immune functions, such as phagocytosis, antibody-dependent cell cytotoxicity, and transcytosis [23]. The vomeronasal type 1 receptor is a G-protein-coupled receptor in olfactory bulbs [24]. These binding partners suggest that peptide Lv may have diverse functions, which need to be investigated.

To confirm that peptide Lv binds to VEGFR2, co-immunoprecipitation assays were performed using chicken embryonic heart lysates [19]. Anti-Lv can pull down VEGFR2, and an antibody specific against VEGFR2 (anti-VEGFR2) is also able to pull down peptide Lv, confirming that peptide Lv and VEGFR2 are binding partners [19]. Furthermore, treatments with peptide Lv in cultured cardiomyocytes increase the phosphorylation of VEGFR2, ERK, and protein kinase C (PKC), the latter two being downstream molecules of VEGFR2 signaling [19]. Thus, peptide Lv activates VEGFR2 and stimulates downstream signaling.

Peptide Lv also increases LTCC current densities in cultured cardiomyocytes [19]. This augmentation of LTCC current densities and phosphorylation of VEGFR2 is attenuated by 4-(2-(4-(3-phenylpyrazolo [1,5-a]pyrimidin-6-yl)phenoxy)ethyl)morpholine (DMH4), a potent VEGFR2 inhibitor [19]. These findings show that LTCC augmentation by peptide Lv is, in part, due to its binding to VEGFR2. Since VEGF binding to VEGFR2 is known to promote angiogenesis by stimulating signaling in vascular endothelial cells [22], it poses the question as to whether peptide Lv is also an angiogenic factor.

## 3. Vascular Endothelial Cells and Angiogenesis

**Figure 3:**
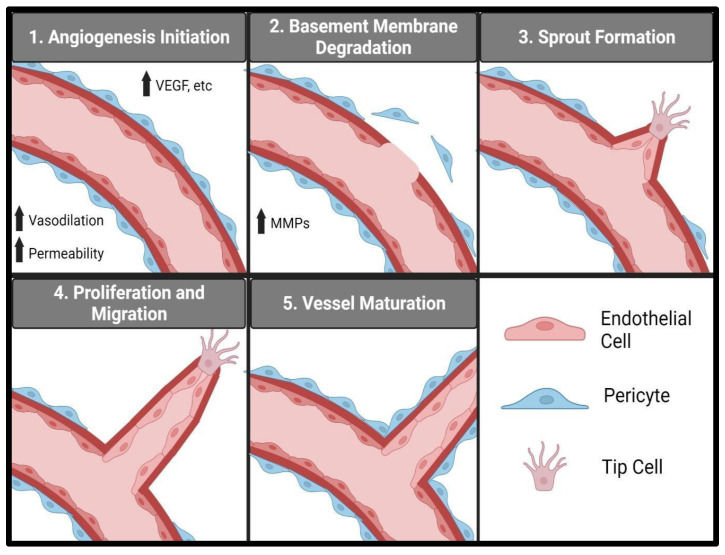
Stages of vascular endothelial cell-mediated angiogenesis. The first stage of angiogenesis is initiated by the reaction to various factors, including VEGF. Local vasodilation and increased permeability occur in response to VEGF. An increase in MMPs, followed by basement membrane degradation ensues. A sprout then forms, followed by the proliferation and migration of endothelial stalk cells, led by a tip cell. Once the vessel tube is formed, the vessel will then mature through its association with pericytes. Created by Biorender.com.

Vascular endothelial cells are essential in angiogenesis, and various endothelial processes, from proliferation, migration, to sprouting, are needed to initiate and promote angiogenesis [25,26,27,28,29,30]. Specialized vascular endothelial cells, called tip cells, determine the formation of new blood vessels by responding to growth factors, such as VEGF [31]. There are several major stages in terms of angiogenesis and neovascularization (Figure 3): first the breakdown of the extracellular matrix and the initiation of migration and invasion of the tip cells occur [31]; next, the endothelial cells proliferate and migrate, followed by tube formation [32]; subsequent stages involve the maturation of the newly formed vessels and pericyte association [33].

The first stage of angiogenesis involves the breakdown of the vascular basement membrane and the degradation of the extracellular matrix, followed by the formation of a sprout from the existing vasculature [28,32]. An increase in vascular permeability in terms of the pre-existing capillaries is needed during the early stages of angiogenesis [34,35]. Several angiogenic factors, including VEGF, are known to increase vascular permeability and are typically upregulated during development, wound healing, or pathological conditions causing angiogenesis [4,9,35,36]. Chronic inflammation involving increased proinflammatory cytokines is known to cause vascular permeability and potential pathological angiogenesis [37]. In addition, chronic vasodilation is associated with the elicitation of angiogenesis [27,28], since vasodilation increases vascular blood flow and sheer stress on the vessel wall, which leads to further chronic increased vascular permeability [34,38]. Increased vascular permeability allows proteases, such as matrix metalloproteinases (MMPs), to enter the extracellular space and degrade the extracellular matrix [27,28]. MMPs degrade laminin and collagen IV in the basement membrane and various components of the extracellular matrix including collagen, fibronectin, laminin, and gelatin [39]. Once the basement membrane and extracellular matrix are degraded, a new vessel can begin to sprout from the existing vessel. Sprouting relies on two types of endothelial cells, tip cells and stalk cells [26,32]. Tip cells respond to various growth factors, like VEGF, and begin migrating into the extracellular space [32]. Stalk cells follow the tip cells through the extracellular matrix and, eventually, form the vessel tube [32].

After the initial sprout is formed, angiogenesis proceeds to the next stage, where the tip cells migrate into the extracellular space, followed by the proliferation and migration of stalk cells [32,40], which eventually connect with the tip cells to form tube structures [32]. Junction formations between endothelial cells lead to the creation of lumen and blood vessel tubes [32]. Cell adhesion proteins, like vascular endothelial cadherin (VE cadherin), form interactions between neighboring endothelial cells and are required for the polarization of endothelial cells, in which the apical surface faces the lumen and the basolateral side faces the extracellular matrix [41,42].

The final stage of angiogenesis involves the maturation of the newly formed blood vessels, mediated by mural cells, like vascular smooth muscle cells and pericytes [33]. Once endothelial cells form immature tube structures, they will then recruit pericytes to initiate maturation [43]. During maturation, the junctions between endothelial cells are stabilized and the vascular permeability is regulated by the pericytes and the endothelial cells [43]. During angiogenesis, in response to wound healing or development, vessel maturation is regulated in response to metabolic need and environmental cues [27,43]. Any excess blood vessel formation will then be pruned and regressed once the wound is healed or the metabolic demand is satisfied [25,27,28]. Although angiogenesis is needed for development and normal physiological processes, uncontrolled angiogenesis contributes to the pathology of many diseases.

Blood vessels grown from pathological angiogenesis do not mature correctly [4,44]. These vessels are leaky and more permeable than normal mature blood vessels [45]. Abnormal angiogenesis tends to be uncontrolled, involving excessive vascular networks [3,46]. Pathological angiogenesis is thought to be caused by an imbalance in pro-angiogenic and anti-angiogenic signaling [3,47]. Many diseases involving pathological angiogenesis have increased VEGF expression [4,9,46]. A large number of therapies target pro-angiogenic molecules, like VEGF, to combat abnormal neovascularization [46,48,49]. However, these therapies have limitations, as neovascularization recurs and patients become resistant to anti-VEGF therapies [12,13,50]. Thus, finding new therapeutic targets, other than VEGF, is clinically important.

## 4. Peptide Lv and Angiogenesis

As mentioned earlier, VEGFR2 is a binding partner of peptide Lv, so it is likely that peptide Lv is pro-angiogenic [19]. VEGF binding to VEGFR2 promotes the three essential processes for angiogenesis, the proliferation, migration, and sprouting of vascular endothelial cells [4,22,32,47]. Cultured human umbilical vein endothelial cells (HUVECs) treated with peptide Lv, using in vitro colorimetric 3-(4,5-dimethylthiazol-2-yl)-2,5-diphenyltetrazolium bromide (MTT) proliferation assays [19,51], have increased cellular proliferation compared to cells treated with a vehicle. Using in vitro scratch wound healing assays to determine the cell migration rates [52], cultured HUVECs treated with peptide Lv were found to have a faster rate of migration compared to the control treated with a vehicle [51]. Three-dimensional (3D) collagen sprouting/invasion assays were used to determine whether peptide Lv can promote endothelial cell sprouting [26,51]. In this assay, a monolayer of HUVECs was first cultured on a 3D collagen matrix, which contained sphingosine 1-phosphate for guiding and promoting endothelial sprouting [26]. After 24 h, the cultures were fixed and stained with toluidine blue to visualize and quantify the endothelial spouting/invasion [26]. The HUVEC cultures treated with peptide Lv had a significantly higher sprouting density compared to the control [51]. These three assays clearly demonstrate that peptide Lv promotes angiogenesis in vitro.

To confirm that peptide Lv is pro-angiogenic in vivo, chicken chorioallantoic membrane (CAM) angiogenesis assays were used [53]. A CAM is an extraembryonic membrane with a capillary network that acts as a gas exchange membrane for the chicken embryo. On embryonic day 7 (E7) or E8, peptide Lv or the vehicle was carefully dropped onto the CAM surface and covered with a small plastic coverslip. On E11–E12, local vascular images were taken under the coverslips and the vascular areas were measured and analyzed [53]. The areas treated with peptide Lv had increased vascular areas compared to the controls, which were treated with a vehicle (phosphate-buffered saline; PBS) [51]. To determine whether peptide Lv could stimulate ocular neovascularization in early postnatal mice, mouse eyes were injected with peptide Lv or PBS (vehicle control) at postnatal day 7 (P7) [51]. On P12, the retinas were isolated and the retinal microvasculature densities were determined in whole mounted retinas stained with fluorescein isothiocyanate (FITC)-conjugated isolectin B4. Eyes injected with peptide Lv had increased retinal vascular areas compared to the PBS controls [51]. These results demonstrate that peptide Lv is an angiogenic factor in vivo.

## 5. Peptide Lv and Pathological Angiogenesis

The upregulation of peptide Lv is positively correlated with pathological angiogenesis in the retina. Diabetic retinopathy is an ocular disease that involves pathological angiogenesis once it progresses to proliferative diabetic retinopathy [7,54]. Peptide Lv is upregulated in the retinas of human patients with early proliferative diabetic retinopathy, dogs suffering from type 1 diabetes, and mice with experimental obesity-associated type 2 diabetes [51]. To further verify the involvement of peptide Lv in pathological angiogenesis, two animal models of ocular angiogenesis were used [9].

An oxygen-induced retinopathy (OIR) mouse model was developed to mimic retinopathy of prematurity (ROP), an eye disease occurring in preterm babies that require oxygen support while they are in incubators [55,56,57]. The basis of this disease is that the retinas of premature babies go from a lower oxygen environment in the womb to a relatively high oxygen environment in an incubator. The excess oxygen causes an initial vaso-regression and forms a vaso-obliteration zone in the retina. As the retinas mature, involving increased metabolic needs, they become hypoxic due to the vaso-regression, stimulating ocular angiogenesis [55]. The pathological angiogenesis in ROP is abnormal and uncontrolled [55]. In the OIR model, mouse pups are placed in a high oxygen chamber (75% oxygen) on P7 to simulate the relatively high oxygen environment that premature babies are exposed to. During this phase, the retinal vessels regress and a vaso-obliteration zone forms. On P12, the mouse pups are returned to normal room air. At this point, the retinas are subject to relatively hypoxic conditions compared to 75% oxygen, stimulating ocular angiogenesis. To test whether an agent is pro- or anti-angiogenic, an intraocular injection with a testing agent (peptide Lv, anti-Lv, or PBS vehicle) is administered on P12. On P17, the vasculature reaches maximum vascularization, so after the retinas are excised and processed, the level of neovascularization is measured by analyzing the amount of neovascular tufts (areas with high levels of vascular sprouting). The areas of any remaining vaso-obliteration zones are also analyzed. After this point, the retinas are acclimatized to normal room air and the vessels in the neovascular areas regress. Gradually, the retinal vasculature returns to a relatively normal state by P25 [57].

Interestingly, the retinas isolated from OIR mice on P17 displayed a significant increase in the mRNA level of peptide Lv compared to the P17 mice without OIR [51], similar to the upregulated VEGF in OIR mouse retinas [58]. Blocking peptide Lv with anti-Lv increases the OIR-associated vaso-obliteration areas compared to the PBS controls, suggesting that blocking peptide Lv will reduce pathological neovascularization [51]. Intraocular injections with peptide Lv reduce OIR vaso-obliteration areas and increase the level of neovascular tufts, suggesting that peptide Lv promotes pathological angiogenesis [51]. To confirm the role of peptide Lv in pathological neovascularization, a peptide Lv knockout mouse model was developed, using CRISPR-Cas9 to delete exon 2 from the *Vstm4* gene [51]. The vaso-obliteration zone was greater and the level of neovascular tufts was less in homozygous (PLv−/−) and heterozygous (PLv+/−) mutant peptide Lv mice compared to the control (PLv+/+), after OIR was elicited [51]. These data provide evidence that peptide Lv is involved in pathological neovascularization.

Laser-induced choroidal neovascularization (CNV) in animal eyes is a model that mimics wet age-related macular degeneration (AMD), another ocular disease involving pathological neovascularization [59]. Wet AMD is associated with abnormal blood vessel growth in aging retinas, especially in the macula area [60], because of increased inflammation and VEGF expression [59]. Unlike the OIR model that examines neovascularization in young animals, the CNV model examines neovascularization in adult animals [59]. In this model, a laser is used to burn the Bruch’s membrane at the back of the retina, which leads to inflammatory responses and the subsequent neovascularization needed for wound healing [59]. Immediately after laser photocoagulation, a testing agent is injected intraocularly. One week after the laser burns were made, the level of vascular leakage from the newly formed blood vessels and the areas/volumes of CNV-elicited neovascularization can be determined using fundus fluorescein angiography, immunostaining with isothiocyanate FITC-dextran on whole mounted retinas, or optical coherence tomography (OCT) [59]. Intraocular injections of anti-Lv after the laser burns were made reduce the severity of the leakage, compared to mice injected with the vehicle (PBS). This reduction in leakage severity suggests a decrease of leaky vessels [51]. Anti-Lv also reduces the level of neovascularization at the lesion sites and the size of the lesions [51]. Thus, peptide Lv is clearly involved in pathological angiogenesis in both OIR and CNV animal models [51].

Furthermore, peptide Lv is synergistic with VEGF in regard to promoting endothelial proliferation [51]. With suboptimal concentrations of peptide Lv and VEGF, which are not able to enhance endothelial cell proliferation when treated singularly, concurrent administration in cultured HUVECs significantly increased endothelial cell proliferation. These findings suggest that in pathological conditions, both VEGF and peptide Lv might be upregulated to further increase neovascularization or even cause recurrent neovascularization. Thus, peptide Lv and VEGF might need to be targeted concurrently for the application of more effective treatments against diseases with pathological angiogenesis.

## 6. VEGF, Endothelial Cells, and Vasodilation

VEGF binding to VEGFR2 not only stimulates angiogenesis, but also elicits endothelial cell-dependent vasodilation [61]. VEGF-elicited vasodilation occurs in a dose-dependent manner, which is attenuated completely by L-NG-Nitroarginine methyl ester (L-NAME), a nitric oxide synthase inhibitor, so VEGF-dependent vasodilation is nitric oxide dependent [61]. As previously stated, chronic vasodilation occurs during the initial stages of angiogenesis [27,28]. Furthermore, chronic vasodilation of existing vessels is associated with increased neovascularization in diseases like proliferative diabetic retinopathy [62,63,64]. The use of vasodilators is associated with an increased incidence of wet AMD [62]. Vasodilation occurs through complex signaling involving vascular smooth muscle cells and endothelial cells [65,66,67]. The removal or inhibition of constriction signals causes vascular smooth muscle cells to relax, allowing vessel lumen expansion [67,68]. Although smooth muscle cells can directly cause vasodilation, signals from endothelial cells play an important role in vasomotor control [66].

When stimulated by agonists or shear stress, endothelial cells can release vasodilators, like nitric oxide [65,69,70,71] or prostacyclin [72], into the intercellular space between endothelial cells and smooth muscle cells. Shear stress, acetylcholine, and cytokines stimulate the production of nitric oxide through endothelial nitric oxide synthases [70,71]. Nitric oxide diffuses to the smooth muscle cells, where it activates soluble guanylyl cyclase to increase the level of cyclic guanosine monophosphate (cGMP), which leads to the phosphorylation of various smooth muscle relaxation signaling pathways [70,71]. Endothelial cyclooxygenase enzymes (COXs) and prostacyclin synthase produce prostaglandins in the endothelium in response to damage, shear stress, and cytokines [72]. Prostaglandins bind to prostaglandin receptors on smooth muscle cells, which leads to the activation of adenylate cyclase [73]. The increased cAMP levels from adenylate cyclase activation activates protein kinase A, leading to the activation of smooth muscle cell relaxation signaling pathways [73].

Another way endothelial cells can elicit vasodilation is through hyperpolarization [74,75,76]. Vasodilation through endothelial-dependent hyperpolarization still occurs even when the synthesis of nitric oxide or prostaglandin is blocked [77]. Endothelial hyperpolarization leads to smooth muscle hyperpolarization [78], which causes smooth muscle cell relaxation by closing the calcium channels and preventing Ca^+2^ influx. Without Ca^+2^ influx, the smooth muscle will not be able to contract, since Ca^+2^ needs to bind to calmodulin to activate myosin light chain kinase and promote smooth muscle contraction [79].

Several processes allow hyperpolarized endothelial cells to trigger smooth muscle cells to hyperpolarize and relax. Myoendothelial gap junctions (MEJs) are specific gap junctions that directly link endothelial cells and smooth muscle cells through the internal elastic lamina [80]. Structurally, MEJs resemble gap junctions in the nervous system [81] and are primarily found in resistance vessels to regulate blood flow and blood pressure [80]. MEJs can directly propagate electrical signals from endothelial cells to neighboring smooth muscle cells, causing them to hyperpolarize [82,83]. Second messengers, like Ca^+2^, and vasodilatory molecules are transmitted between endothelial and smooth muscle cells through MEJs [82,84,85].

Endothelial cells can release factors called endothelial-derived hyperpolarizing factors (EDHF), including potassium (K^+^) ions, that lead to smooth muscle hyperpolarization [74,78,86]. During endothelial hyperpolarization, K^+^ ions flow out of the cells and cause a small increase in extracellular K^+^ that activates inward-rectifying K^+^ (Kir) channels and sodium/potassium pumps (Na^+^/K^+^-ATPase) on smooth muscle cells [87,88,89,90]. Normally, Kir channels can have both inward and outward currents, but the outward currents are normally blocked [89,91]. Small increases in extracellular K^+^ unblock the outward currents from Kir channels, enabling K^+^ efflux from the smooth muscle Kir channels, leading to their hyperpolarization and relaxation [89]. Na^+^/K^+^ pumps maintain the ionic gradients of K^+^ and Na^+^ in and out of the cells. When there is a small increase in extracellular K^+^, the pumps unequally send three Na^+^ ions out and two K^+^ ions in, causing an overall hyperpolarizing effect in smooth muscle cells [87].

Epoxyeicosatrienoic acids (EETs) are another possible EDHF that cause vasodilation [92]. EETs are products of membrane-bound cytochrome P450 epoxygenases. Endothelial cells produce and release EETs in response to shear stress or vasodilatory agonists, like bradykinin [92]. EETs cause hyperpolarization through the activation of calcium-dependent potassium (K_Ca_) channels in endothelial cells and smooth muscle cells [93,94]. K_Ca_ channels are the primary K^+^ channels involved in hyperpolarization, by releasing K^+^ ions when opened [95]. K_Ca_ activity can be augmented by modulating the level of intracellular calcium [96]. EETs can activate channels like transient receptor potential (TRP) channels to increase the influx of calcium, which further activates K_Ca_ channels [97].

## 7. Peptide Lv and Vasodilation

Since peptide Lv can bind to VEGFR2 and stimulate angiogenesis, the question becomes can peptide Lv also elicit vasodilation. Using an ex vivo vasomotor activity assay [61,98], with freshly isolated porcine coronary and retinal arterioles, peptide Lv elicits dose-dependent vasodilation [51]. Interestingly, while VEGF (via VEGFR2)-elicited vasodilation is completely blocked by the nitric oxide synthase inhibitor L-NAME, peptide Lv-elicited vasodilation is only partially blocked by L-NAME, suggesting that peptide Lv has VEGF/VEGFR2/nitric oxide-dependent and -independent actions [51]. This finding emphasizes the importance of studying peptide Lv, as its bioactivities during vasodilation and angiogenesis have VEGF-independent components. Since peptide Lv is involved in pathological angiogenesis as described previously, its VEGF-independent actions might explain the resistance of some patients to anti-VEGF therapies.

## 8. Vasodilation and Angiogenesis: Involvement of Endothelial Ion Channels

**Figure 4:**
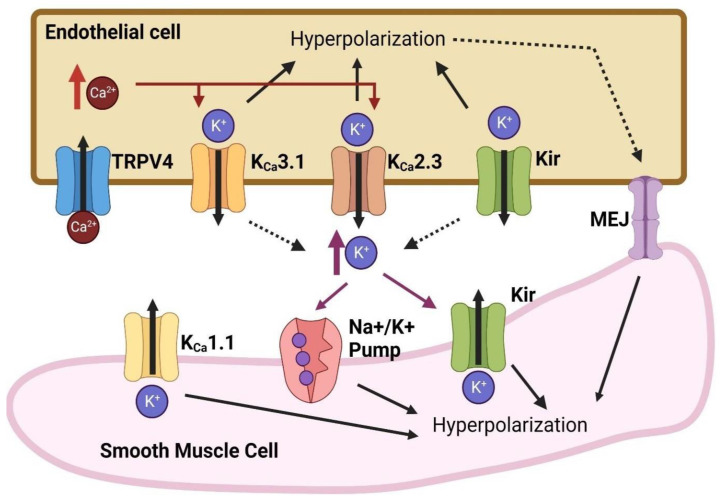
Various ion channels are involved in hyperpolarization. Endothelial K^+^ channels, such as K_Ca_3.1, K_Ca_2.3, and Kir channels, release K^+^, leading to endothelial hyperpolarization. The electrical signal can be propagated to smooth muscle cells through myoendothelial junctions (MEJs). K^+^ efflux into the myoendothelial space can activate smooth muscle Kir channels and Na^+^/K^+^ pumps, leading to smooth muscle hyperpolarization. TRPV4 channel opening leads to increased calcium influx that can activate K_Ca_ channels. Created with Biorender.com.

As stated previously, hyperpolarization of endothelial cells may cause vasodilation and initiate angiogenesis [74,75,76]. Various ion channels are involved in endothelial cell hyperpolarization (Figure 4). The outflow of K^+^ ions, through various K^+^ channels, hyperpolarizes endothelial cell membranes [99]. Calcium-dependent K^+^ (K_Ca_) channels, especially small-conductance K_Ca_ (K_Ca_2.x) and intermediate-conductance K_Ca_ (K_Ca_3.1), contribute to endothelial hyperpolarization, as blocking these channels will prevent vasodilator-induced hyperpolarization [95,100]. Other K^+^ channels including inward-rectifying K^+^ (Kir), ATP-sensitive K^+^ (K_ATP_) channels, and transient receptor potential (TRP) channels have also been associated with endothelial hyperpolarization [101,102,103,104,105]. TRP channels are not cation specific and are activated by different stimuli [103]. In terms of endothelial hyperpolarization, the opening of TRP channels allows Ca^+2^ influx, which activates endothelial K_Ca_ channels [101,103,104,106]. Furthermore, ion pumps, such as Na^+^/K^+^-ATPase, facilitate endothelial hyperpolarization due to the unequal transfer of Na^+^ and K^+^ ions against their concentration gradients, which results in more positive ions leaving than entering [87,107].

### 8.1. Calcium-Dependent Potassium (K_Ca_) Channels

Calcium-dependent potassium (K_Ca_) channels are known to participate in hyperpolarization through outward K^+^ currents in vascular endothelial cells [95,108,109]. There are three subtypes of K_Ca_: large-conductance K_Ca_ (K_Ca_1.1 or BKCa), intermediate-conductance K_Ca_ (K_Ca_3.1 or IKCa), and small-conductance K_Ca_ (K_Ca_2.x or SKCa) [110]. A certain level of intracellular Ca^+2^ or influx of Ca^+2^ is needed for the opening of K_Ca_ channels [111]. K_Ca_1.1 channels are the major K^+^ channels that hyperpolarize smooth muscle cells [75], but they are not as widely expressed in endothelial cells, so blocking K_Ca_1.1 does not affect endothelium-dependent vasodilation [112]. K_Ca_2.3 and K_Ca_3.1 are the major K_Ca_ channels expressed in vascular endothelial cells [95,100,113]. In contrast to K_Ca_1.1 channels, K_Ca_2.3 and K_Ca_3.1 channels are more sensitive to Ca^+2^ concentration changes and their opening occurs independent of voltage [96,111]. Pharmacologically blocking either of these channels attenuates vasomotor responses to endothelium-dependent hyperpolarizing factors [95,100,113]. The genetic deletion of these channels in mice causes a reduction in vasodilation in response to endothelial-dependent hyperpolarization [114,115]. In addition, K_Ca_3.1 channels are involved in nitric-oxide-independent vasodilation [116]. Furthermore, the activation of K_Ca_ channels promotes cell proliferation and migration in various cell types, from vascular smooth muscle cells to vascular endothelial cells [117,118], and more specifically, K_Ca_2.3 and K_Ca_3.1 channels are involved in endothelial cell proliferation [117,118,119], which is essential for angiogenesis [119]. Pharmacologically blocking the K_Ca_3.1 channel attenuates growth factor-induced angiogenesis in vivo and in vitro [117].

### 8.2. Inward-Rectifying Potassium (Kir) Channels

Inward-rectifying potassium (Kir) channels are voltage dependent, where at resting membrane potential, magnesium (Mg^+2^) and polyamines block the outward flow of K^+^ [88,89,120]. When the membrane potential is hyperpolarized, Mg^+2^ and polyamines dissociate, which allows K^+^ efflux [88]. Kir channel activity is also dependent on the extracellular K^+^ concentration, an increase of which enhances the outward current of Kir channels [121]. A phosphatidylinositol 4,5-bisphosphate (PIP2) interaction is necessary for Kir opening, as the depletion of PIP2 significantly reduces Kir activity [122].

Kir channels are expressed in both endothelial and smooth muscle cells [88], participate in the hyperpolarization of vascular endothelial cells, and stimulate Ca^+2^ influx [123]. The genetic deletion of these channels inhibits vasodilation [123].

### 8.3. ATP-Sensitive Potassium (K_ATP_) Channels

ATP-sensitive potassium (K_ATP_) channels are comprised of four pore-forming Kir subunits (Kir6.1 or Kir6.2) and four large regulatory sulfonylurea receptor subunits (SUR1, SUR2A, or SUR2B) [124]. Each Kir subunit has an ATP-binding site and, when bound, the channel shifts to a more closed state [125]. Each SUR subunit contains two adenosine nucleotide binding sites [91] and, when ADP is bound, one site will dimerize with another nucleotide binding site and favor K_ATP_ channel opening [91,126]. K_ATP_ channels are expressed in both smooth muscle and endothelial cells and are involved in endothelial hyperpolarization. Blocking K_ATP_ channels reduces endothelial hyperpolarization caused by hypoxia-associated reperfusion [99,127]. Mice with an endothelial-specific knockout of the Kir6.1 subunit develop hypertension and experience dysfunctional vasomotor activity, suggesting that it has a role in maintaining blood pressure homeostasis [128,129]. In addition, the activation of endothelial K_ATP_ channels stimulates angiogenesis in vivo and in vitro [130].

### 8.4. Transient Receptor Potential Cation (TRP) Channels

The transient receptor potential (TRP) family of ion channels can be divided into six subfamilies, according to their sequence homology: TRPA (ankyrin), TRPC (canonical), TRPM (melastatin), TRPML (mucolipin), TRPP (polycystin), and TRPV (vanilloid) [103]. They are nonselective cation channels with varying cation specificity, depending on the subtype [103,131,132], and they open as a result of membrane depolarization [103,133] or other various stimuli, including mechanical force [132,134], electrical changes [103,131], ligand binding [103,132], and changes in temperature [103,131,132]. Shear stress from increases in blood flow stimulates the opening of endothelial TRPV4 channels [135].

TRP channels can play a role in the control of vascular tone and angiogenesis [103,106,136]. Over 20 different types of TRP channels are expressed in vascular endothelial cells and several of them regulate intracellular Ca^+2^ in regard to vasomotor activities [104]. TRPV4 has been extensively studied for its role in endothelium-dependent vasodilation [101]. TRPV4 knockout in mice impairs acetylcholine-induced and flow-mediated vasodilation [105,137]. TRPV4 channels are closely involved in the stimulation of endothelial K_Ca_3.1 and K_Ca_2.3 channels, as activating TRPV4 channels enhances the currents in K_Ca_ channels [138]. Several TRP channels are involved in angiogenesis [139,140,141]. The knockout of different TRPC channels results in disrupted tube formation [139,140]. The knockout of TRPV4 reduces endothelial migration and sprouting [141]. The activation of TRPV4 promotes endothelial cell proliferation and angiogenesis [142].

## 9. Peptide Lv Augments Endothelial K_Ca_3.1 Channels

One of the initial steps in endothelial cell-dependent vasodilation or angiogenesis is hyperpolarization of the endothelial cell membrane [25,116]. Treatments with peptide Lv for at least 3 h cause the hyperpolarization of HUVECs [14]. This peptide Lv-induced 439 endothelial hyperpolarization is mediated by K_Ca_3.1 channels. HUVECs treated with peptide Lv show a decrease in K_ATP_ channels (Kir6.1), without affecting K_Ca_2.3 or TRPV4 channels. However, peptide Lv increases the mRNA and protein expression of K_Ca_3.1 channels, as well as augments the K_Ca_3.1 current density in HUVECs, so among the ion channels that are able to hyperpolarize endothelial cells, peptide Lv-elicited endothelial hyperpolarization occurs specifically through the K_Ca_3.1 channel. In addition, peptide Lv-elicited endothelial proliferation is blocked by the K_Ca_3.1 inhibitor TRAM-34 [14].

Peptide Lv not only increases the mRNA and protein expression of K_Ca_3.1 in vascular endothelial cells, but also promotes the trafficking of K_Ca_3.1 from the cytoplasm to the plasma membrane through the activation of two parallel signaling pathways, mitogen-activated protein kinase 1 (MEK1)-extracellular signal-regulated kinase (ERK) and phosphoinositide 3 kinase (PI3K)-protein kinase B (Akt) [143]. The inhibition of either pathway blocks peptide Lv-elicited endothelial hyperpolarization and increases the K_Ca_3.1 current density. However, the inhibition of MEK1-ERK does not affect peptide Lv-elicited phosphorylation (activation) of PI3K-Akt and, vice versa, in cultured vascular endothelial cells [143]. Hence, peptide Lv-elicited hyperpolarization of vascular endothelial cells occurs through the increased expression of K_Ca_3.1 and protein trafficking of K_Ca_3.1 into the plasma membrane, which is, in part, the underlying mechanism as to how peptide Lv promotes endothelium-medicated angiogenesis.

## 10. Scientific and Clinical Relevance of Peptide Lv

Peptide Lv is upregulated in the retinas of patients with diabetic retinopathy, diabetic animals, and animals with OIR, which indicates that it is indeed involved in pathological angiogenesis. As a promoter of vascular endothelial cell proliferation, migration, and sprouting, peptide Lv has synergistic effects with VEGF [51], implying that in chronic pathological conditions, not only are VEGF and peptide Lv upregulated, but their synergistic effects could further worsen neovascularization. The action of peptide Lv is in part VEGF/nitric oxide-dependent, but peptide Lv also has VEGF-independent action(s), which could be one cause of VEGF resistance in patients treated with anti-VEGF agents.

As peptide Lv is expressed in various organs, there could be other functions of peptide Lv that are yet to be determined. One possibility is the function of peptide Lv in immune systems and inflammatory responses, as it is expressed in the spleen [15,19]. In peptide Lv knockout mice, the lipopolysaccharide-induced inflammatory response was more severe compared to the control (wild type) [144]. As peptide Lv can augment L-type voltage-gated calcium channels (LTCCs) in neurons and cardiomyocytes and it is indeed expressed in the brain and heart, it is possible that through modulating LTCCs peptide Lv has other regulatory functions in the brain and heart. Aging generally causes decreased vasculature, and compared to wild-type mice, peptide Lv knockout mice at 12 months old have significantly lower retinal vasculature densities [51], which indicates that the loss of peptide Lv worsens vascular degeneration. In patients with Alzheimer’s disease, vascular density is decreased in the brain, with diminished angiogenic functions [145]. Much of the memory loss experienced by Alzheimer’s patients is due to the degeneration of the hippocampus [146], and peptide Lv is highly expressed in the hippocampus and cerebral cortex [15]. Thus, one could speculate that the loss of peptide Lv due to aging might contribute to dementia and possibly the pathogenesis of Alzheimer’s disease. Since peptide Lv is widely expressed in the body, understanding its bioactivities, functions, and molecular mechanisms of action will open doors for the future development of new therapeutics for treating or preventing diseases.

## 11. Potential Therapeutics Targeting Peptide Lv

To date, peptide Lv is known to impact various cell types, including photoreceptors, cardiomyocytes, and vascular endothelial cells (Table 1). The most understood function of peptide Lv is its ability to promote angiogenesis and vasodilation, in part, through the activation of VEGFR2. An antibody against peptide Lv, anti-Lv, can inhibit pathological neovascularization in animal models, mimicking retinopathy of prematurity (OIR) and wet AMD (laser-induced CNV). Furthermore, peptide Lv is upregulated in the retinas of patients with diabetic retinopathy, diabetic animals, and animals with OIR, indicating that it is indeed involved in pathological angiogenesis. Since peptide Lv has synergistic effects with VEGF in promoting angiogenesis, it implies that in chronic pathological conditions, not only are VEGF and peptide Lv upregulated, but their synergism might further worsen neovascularization. The action of peptide Lv is in part VEGF/nitric oxide-dependent, but peptide Lv also has VEGF-independent action(s), which could be one cause of VEGF resistance in patients treated with anti-VEGF agents. The synergism between VEGF and peptide Lv might be an underlying reason for recurring neovascularization in these diseases. Hence, therapies blocking peptide Lv might be used to treat diseases involving pathological angiogenesis, including diabetic retinopathy and wet AMD. Furthermore, in conditions such as ischemia and wound healing, peptide Lv or its analogs could be applied where angiogenesis is needed. Future studies into the mechanisms and plausibility of treatments targeting peptide Lv are needed.

## Figures and Tables

**Table 1:** Bioactivities of peptide Lv.

Cell/Tissue Types	Bioactivities	References
Photoreceptors (chicken embryos)	Increases activation/phosphorylation of adenylate cyclase and ERK; Increases mRNA and protein expressions of Cav1.2 (LTCC−1C) and Cav1.3 (LTCC−1D) and LTCC current densities.	Shi, et al., 2012. [15] PMID: 22912796
Cardiomyocytes (chicken embryos)	Increases protein expression and current densities of LTCCs; Increases phosphorylation/activation of VEGFR2; Activates MAPK, PKC, and tyrosine kinase.	Shi, et al., 2015. [19] PMID: 25698653
Human umbilical vein endothelial cells (HUVECs)	Binds to VEGFR2; Increases activation/phosphorylation of VEGFR2, ERK, and Akt (at s473); Promotes cell proliferation, migration, and sprouting; Causes cell hyperpolarization; Increases mRNA and protein expressions of Kcnn4/K_Ca_3.1 and K_Ca_3.1 current densities; Promotes K_Ca_3.1 protein trafficking from cytosol to cell membranes.	Shi, et al., 2015. [19] PMID: 25698653 Shi, et al., 2019. [51] PMID: 31698979 Pham, et al., 2022. [14] PMID: 36282858 Pham, et al., 2023. [143] PMID: 37371121
Human retinal microvascular endothelial cells (HRMECs)	Promotes cell proliferation; Synergistic with VEGF; Increases the protein expression of K_Ca_3.1; Increases activation/phosphorylation of ERK and Akt (at s473).	Shi, et al., 2019. [19] PMID: 31698979 Pham, et al., 2022. [14] PMID: 36282858 Pham, et al., 2023. [143] PMID: 37371121
Pig coronary and retinal arterioles (ex vivo)	Elicits dose-dependent vasodilation.	Shi, et al., 2019. [19] PMID: 31698979
Chicken chorioallantoic membrane (ex ovo)	Promotes angiogenesis.	Shi, et al., 2019. [19] PMID: 31698979
Mouse retina (in vivo)	Promotes angiogenesis and pathological neovascularization.	Shi, et al., 2019. [19] PMID: 31698979
Mice (whole animals)	Dampens lipopolysaccharide-induced inflammatory responses.	Mukai et al., 2021. [144] PMID: 34386509
